# A Powerful New Quantitative Genetics Platform, Combining *Caenorhabditis elegans* High-Throughput Fitness Assays with a Large Collection of Recombinant Strains

**DOI:** 10.1534/g3.115.017178

**Published:** 2015-03-13

**Authors:** Erik C. Andersen, Tyler C. Shimko, Jonathan R. Crissman, Rajarshi Ghosh, Joshua S. Bloom, Hannah S. Seidel, Justin P. Gerke, Leonid Kruglyak

**Affiliations:** * Department of Molecular Biosciences, Northwestern University, Evanston, Illinois 60208; † Lewis-Sigler Institute for Integrative Genomics, Princeton University, Princeton, New Jersey 08544; ‡ Howard Hughes Medical Institute, Departments of Human Genetics and Biological Chemistry, David Geffen School of Medicine, U.C.L.A., Los Angeles, California 90095

**Keywords:** *C. elegans*, QTL mapping, high-throughput phenotyping, fitness assays

## Abstract

The genetic variants underlying complex traits are often elusive even in powerful model organisms such as *Caenorhabditis elegans* with controlled genetic backgrounds and environmental conditions. Two major contributing factors are: (1) the lack of statistical power from measuring the phenotypes of small numbers of individuals, and (2) the use of phenotyping platforms that do not scale to hundreds of individuals and are prone to noisy measurements. Here, we generated a new resource of 359 recombinant inbred strains that augments the existing *C. elegans* N2xCB4856 recombinant inbred advanced intercross line population. This new strain collection removes variation in the neuropeptide receptor gene *npr-1*, known to have large physiological and behavioral effects on *C. elegans* and mitigates the hybrid strain incompatibility caused by *zeel-1* and *peel-1*, allowing for identification of quantitative trait loci that otherwise would have been masked by those effects. Additionally, we optimized highly scalable and accurate high-throughput assays of fecundity and body size using the COPAS BIOSORT large particle nematode sorter. Using these assays, we identified quantitative trait loci involved in fecundity and growth under normal growth conditions and after exposure to the herbicide paraquat, including independent genetic loci that regulate different stages of larval growth. Our results offer a powerful platform for the discovery of the genetic variants that control differences in responses to drugs, other aqueous compounds, bacterial foods, and pathogenic stresses.

The nematode *Caenorhabditis elegans* is emerging as a powerful model to connect quantitative traits to genetic variants (Gaertner and Phillips 2010). These quantitative trait genes are easily identified because of favorable attributes of the organism, including a selfing hermaphroditic lifestyle, the ability to generate large numbers of recombinant offspring, the ability to cryopreserve and revive strains indefinitely, and ample phenotypic variation.

To facilitate quantitative genetic mapping, a variety of strain resources have been created. Two crosses between the laboratory-adapted Bristol (N2) strain and a wild strain from Hawaii (CB4856) are available, comprising one panel of 200 F_2_ recombinant lines (Li *et al.* 2006) and one panel of 239 F_10_ advanced intercross lines (Rockman and Kruglyak 2009). These two sets of strains and a set of nearly isogenic lines, or NILs (Doroszuk *et al.* 2009), are proving to be powerful community resources to connect phenotype to genotype because they are freely available and can be used indefinitely. Additionally, a growing collection of wild *C. elegans* strains facilitates genome-wide association studies (Andersen *et al.* 2012).

Unfortunately, these recombinant inbred lines and NILs are not optimal for quantitative trait mapping for two reasons. First, before the creation of these strain resources, the N2 strain underwent a long period of laboratory adaptation. During this time, alleles accumulated that promoted survival under laboratory conditions, including an allele of the *npr-1* gene that encodes a neuropeptide receptor with large physiological and behavioral effects (Andersen *et al.* 2014). Because of the widespread effects of this allele under laboratory conditions, the *npr-1* gene is often detected as causal when mapping quantitative traits in any panel of recombinant strains that include the N2 version of the gene. Second, both panels of recombinant strains constructed between N2 and CB4856 have skewed allele frequencies on the left arm of chromosome I (Rockman and Kruglyak 2009; Li *et al.* 2006). This skew is the result of a genetic incompatibility that causes strains without protection of the N2-provided *zeel-1* gene to be killed by the toxic effects of the N2-provided *peel-1* gene (Seidel *et al.* 2011; 2008). Mapping panels that eliminate *npr-1* variation and the allele frequency skew are highly desirable.

To identify many of the alleles underlying complex traits, the phenotypes of a large number of independent strains need to be measured accurately. Recent efforts in yeast have shown that cross populations as large as one thousand individuals can identify most of the loci that contribute additively to phenotypic differences in a population (Bloom *et al.* 2013). The *C. elegans* community requires a large panel of recombinant strains to increase the statistical power to detect more alleles of ever decreasing effect sizes. However, the majority of assays used in this model organism are optimized for the measurement of a small number of strains, because most research groups focus on the laboratory strain. To connect phenotype to genotype for complex traits, we require a larger panel of recombinant strains and high-throughput assays to measure the phenotypes of a large number of independent strains.

Here, we describe the creation of 359 additional N2xCB4856 recombinant inbred advanced intercross lines (RIAILs) and the optimization of high-throughput fitness assays to measure phenotypes from hundreds of independent strains in parallel. The strains in our new RIAIL panel all have the Hawaiian version of *npr-1* and have a transposon insertion into the *peel-1* gene encoding the incompatibility toxin. These strains do not have the large phenotypic differences caused by variation in the laboratory-adapted allele of *npr-1* and have a reduced allele frequency skew at the incompatibility region on chromosome I. Additionally, we took advantage of the large particle nematode sorter made by Union Biometrica to measure the numbers, lengths, optical densities, and fluorescence of large populations of offspring derived from over a hundred independent lines per day. We optimized liquid growth conditions to massively scale the assays and to significantly reduce environmental heterogeneity normally present in standard laboratory agar plate assays. Using these high-throughput fitness assays, we measured a large number of parameters in our new panel of 359 RIAILs in control conditions and after exposure to the herbicide paraquat. We identified quantitative trait loci (QTL) that individually explain as little as 4% of trait variance, showing the statistical power of this large mapping panel. We believe that this new strain resource, along with a scalable and accurate high-throughput phenotyping platform, will further strengthen *C. elegans* as a powerful organism to understand the functional consequences of natural variation in metazoans.

## Materials and Methods

### Strains

Animals were cultured and all crosses performed at 20° with the bacterial strain OP50 on modified nematode growth medium containing 1% agar and 0.7% agarose to prevent burrowing of strains with the CB4856 version of *npr-1*. For each assay, strains were grown for at least five generations with no strain entering starvation or encountering dauer-inducing conditions. QX1430 contains the nearly isogenic region *qgIR1 [X*, *CB4856 > N2*, *4,754,307-4,864,273]* with the CB4856 version of *npr-1* and a transposon insertion *ttTi12715*, which reduces the function of *peel-1* rendering the strain compatible with CB4856. Other transposon insertions present in the parent strain IE12715 were outcrossed to N2 and confirmed lost by inverse PCR. Throughout the manuscript and this analysis, QX1430 was used in place of the Bristol strain. The Bristol (N2) strain has many other laboratory-derived variants, including *glb-5*. Because most have unknown effects on the N2 phenotype, we did not and could not correct them all.

### Recombinant inbred line construction by advanced intercross

We constructed RIAILs from reciprocal crosses between QX1430 and CB4856. The reciprocal crosses yielded two classes each of male and hermaphrodite progeny that differed with respect to their mitochondrial and X chromosomes. Each of the four possible crosses among the four F1 classes was performed, yielding two classes of hermaphrodite and two classes of male F2 progeny. Each of the four possible crosses from the F2 classes was then performed. F3 progeny were randomly single-pair mated each generation for 10 generations. Each pair contributed equally to each generation (Rockman and Kruglyak 2008).

Each cross plate constituted a single-pair mating between one male and one hermaphrodite. With each generation, a proportion of the crosses failed due to poor male mating. An absence of males in the population of progeny indicated the failure of a cross. Additionally, crosses failed when animals crawled to the edge of the plate and were lost. The proportion of failed crosses was reduced through preparation of three redundant plates for each cross. One line resulting from each cross was then propagated for ten generations through the random selection and selfing of a hermaphrodite. Each randomly selected hermaphrodite was picked with twofold redundancy to reduce the loss of lines during the selfing phase. These strains are available upon request from the Andersen laboratory.

### Genotyping recombinant inbred lines

After RIAIL strains were generated, we froze a strain copy and collected DNA in parallel from the same culture plates. We collected and cleaned DNA using the DNeasy Blood and Tissue Kit (QIAGEN) from populations of animals grown on three 10-cm plates seeded with X1666 *E. coli* bacteria. The strains were genotyped using the Illumina GoldenGate assay as described previously (Rockman and Kruglyak 2009). Raw data from the Illumina reader were read into and processed in R. We used normal mixture modeling via the expectation-maximization (EM) algorithm in the *mclust* R package to determine the genotype of each RIAIL at each single-nucleotide polymorphism (SNP) assuming two groups. This method assigned genotype data for all 1536 SNPs. SNP UCE2-2369 was excluded from analyses because of poor genotyping on the GoldenGate platform. A total of 7017 of 589,824 genotype calls were set to not applicable (NA) because of poor differentiation into either an N2 or CB4856 call. To keep our new RIAIL panel compatible with the previous panel, we selected the same 1454 SNPs genotyped in the original set of N2xCB4856 RIAILs and processed those SNPs. SNPs with allele frequencies greater than 65% N2 or 60% CB4856 were set to NA. We imputed missing and poorly assigned genotypes using the hidden Markov model implemented in the *qtl* package in R (Broman *et al.* 2003). Five SNPs (UCE1-1433, UCE5-3001, CE5-218, UCE6-686, UCE6-1285) were set to NA because the allele frequency skew and later imputation made assignment errors. Re-imputation led to uniform recombination maps. Our genotyping code, raw files, processed data (File S1), a new R/qtl cross object, a marker liftover file, and code to merge phenotypes into this cross object can be found on the Andersen lab GitHub page.

### High-throughput fitness assays of fecundity and body size

Animals were grown in 96-well microtiter plates to allow for automated liquid handling to speed assay set up and phenotyping. In each well, the final volume was 50 µL of S medium solution and HB101 bacterial food. HB101 was prepared in large batches (greater than 20 L) to reduce assay-to-assay variability. The bacteria were prepared from cultures grown for 24 hr in Superbroth and then centrifuged to concentrate. HB101 bacteria were resuspended at 20% volume per volume in S medium and frozen at −80° (without glycerol) in 1-mL aliquots. Bacteria were thawed and fed to animals at a final concentration of 2% volume per volume. This amount of food was sufficient to sustain the number of offspring derived from a single fourth larval stage hermaphrodite with food left over at the end of the assay. For any mapping or causality experiment, we used the same HB101 culture to reduce the substantial variation that arises from bacteria grown in independent preparations. Solutions of S medium and bacterial food were prepared and then split for control and paraquat conditions.

After assay plates were prepared, one mid to late fourth larval stage animal was singled using a platinum wire to each 50-µL well. In the plate set up, we separated different genotypes with a wash well containing only S medium to decrease carry-over and mixing of independent genotypes from one well to the next. After all wells were populated with animals, the microtiter plate was sealed with a Breath-Easy film (USA Scientific) and placed in a humidity chamber lined with damp towels, closed, and then sealed with parafilm. We observed less than 5% of the well volume evaporated after four days under these conditions. Humidity chambers were placed into incubators set to 20° and shaking at 180 rpm. These conditions ensured that cultures and bacterial food was constantly mixing so animals would never enter hypoxia nor encounter regions of depleted food in the well. Animals were grown for 96 hr in these conditions and then prepared for measurement on the COPAS BIOSORT (Union Biometrica).

Two minutes before the animals were loaded on the COPAS BIOSORT, 200 µL of M9 with 50 mM sodium azide was added to the wells. The sodium azide kills and straightens the animals to ensure proper measurement of body length as the paralyzed animal passes through the flow cell. The COPAS BIOSORT sheath flow rate was kept constant at 9.8 mL per minute to decrease variability in length measurements as much as possible. Then, 96-well microtiter plates were aspirated using the ReFLx module with the BISORT set to “no-bubble-trap” mode. When the system is run in “no-bubble-trap” mode, all wells from a single microtiter plate can be measured in approximately 25 min and well-to-well contamination is less than 1%, which is further mitigated by the use of wash wells between unique genotypes. Extinction and time of flight minimums were 50 and 20, respectively. Green, yellow, and red photomultiplier tubes were set to 700, 700, and 900, respectively. Signal multipliers were set to 1.0 and signal gains set to 3.0. The COPAS BIOSORT software had Profiler II enabled. Flat comma-separated value files were analyzed using custom scripts in the R statistical computing environment. The COPAS BIOSORT software can not differentiate bubbles from animals as objects pass through the flow cell. For this reason, we trained a support vector machine to differentiate these two types of objects with 99.97% accuracy (Supporting Information, Figure S1). The support vector machine primarily uses optical density raw values to generate a binary linear classifier separating bubbles from animals. The raw data were read in, processed, and plotted using the *COPASutils* R package (Shimko and Andersen 2014). All of the raw and processed data (File S2) can be accessed on the Andersen lab GitHub page. High-throughput assay plates with paraquat were prepared at 1.5 mM paraquat (Chem Service, Inc.) or no paraquat from the same dilution of HB101 and S medium.

### Linkage mapping

A total of 357 RIAILs were phenotyped in the high-throughput assays described previously for both control and paraquat conditions. The phenotype data and genotype data were entered into R and scaled to have a mean of zero and a variance of one for linkage analysis. QTL were detected by calculating logarithm of odds (LOD) scores for each marker and each trait as -n(ln(1-r^2)/2ln(10)), where r is the Pearson correlation coefficient between RIAIL genotypes at the marker and phenotype trait values (Bloom *et al.* 2013). The maximum LOD score for each chromosome for each trait was retained from three iterations of linkage mappings (File S3 and File S4). We randomly permuted the phenotype values of each RIAIL while maintaining correlation structure among phenotypes 1000 times to estimate significance empirically. The ratio of expected peaks to observed peaks was calculated to determine the 5% false discovery rate of LOD 2.97.

Broad-sense heritability was calculated as the fraction of phenotypic variance explained by strain from fit of a linear mixed-model of repeat phenotypic measures of the parents and RIAILs (Brem *et al.* 2005). The total variance explained by each QTL was divided by the broad-sense heritability to determine how much of the heritability is explained by each QTL. Confidence intervals were defined as the regions contained within a 1.5 LOD drop from the maximum LOD score.

## Results

### An expanded collection of RIAILs

*C. elegans* quantitative genetics was accelerated by the creation and use of an advanced intercross recombinant inbred line collection between the N2 (Bristol) and Hawaii (CB4856) strains or N2xCB4856 RIAILs (Rockman and Kruglyak 2009). This collection of strains facilitated the linkage mapping of a large number of diverse traits (Andersen *et al.* 2014; Bendesky *et al.* 2011; 2012; Gaertner and Phillips 2010; Gaertner *et al.* 2012; Ghosh *et al.* 2012; Glater *et al.* 2014; McGrath *et al.* 2009; Palopoli *et al.* 2008; Pollard and Rockman 2013; Reddy *et al.* 2009; Rockman *et al.* 2010; Seidel *et al.* 2008). Despite these results, this RIAIL panel had three distinct disadvantages. First, a genetic incompatibility between the N2 and Hawaii strains led to a large genotype skew on chromosome I, where nearly all of the strains in the panel have the N2 allele. Second, because the cross was performed at 25°, the strain collection had high levels of sickness and some strains were lost. To combat the strain loss, each strain was duplicated after the intercrossing phase to make a panel where half of the population is highly related to the other half. Third, the laboratory-derived allele at *npr-1* has a large effect on the growth and physiology of *C. elegans* because of behavioral changes. These changes do not represent the natural behavior, growth, or physiology of the species (Andersen *et al.* 2014). We set out to augment this existing resource while also correcting these three disadvantages.

To deal with the strain incompatibility and the laboratory-derived allele of *npr-1*, we created a new strain to use as the N2 parent. For the strain incompatibility, the *peel-1* gene is necessary and sufficient to kill animals that do not have a functioning *zeel-1* gene product (Seidel *et al.* 2011). If we eliminate the function of *peel-1*, the strain will be compatible with any other strain, including the Hawaii strain. We took advantage of an existing transposon insertion into the *peel-1* gene, which is predicted to eliminate the function of *peel-1*. We found this strain to be compatible with both the N2 and Hawaii strains (Figure S2). For the laboratory-derived allele of *npr-1*, we used the nearly isogenic region present in *qgIR1*, where the *npr-1* genomic region from N2 is replaced with the equivalent genomic region from the Hawaii strain. This new strain, named QX1430, is compatible with the Hawaii strain and has the natural allele of *npr-1*.

After the creation of QX1430, we performed an advanced intercross identical to the design previously implemented (Rockman and Kruglyak 2009), except we crossed animals at 20° to reduce temperature-sensitive sickness. In parallel, these strains were frozen for permanent storage and DNA prepared for genotyping. We genotyped the strains using the same polymorphic markers as the previous set. We determined the genotypes of the new recombinant inbred line panel using custom scripts described previously (see *Materials and Methods*). Our new collection of RIAILs comprises 359 independent strains. We refer to this panel as a N2xCB4856 panel to indicate the primary genetic background of the QX1430 parental strain.

Surprisingly, we still observed an allele frequency skew on chromosome I that was less pronounced than in the previous N2xCB4856 RIAIL collection (Figure S3) but greater than expected from random drift (Rockman and Kruglyak 2009). We showed that the skew is caused by a reduced but still present genetic incompatibility (Figure S2). These results suggest that other genes besides *peel-1* might weakly contribute to the incompatibility at that genomic location or that the function of *peel-1* is not completely abrogated by the transposon insertion. We also noted that other weaker allele frequency skews on other chromosomes in the original RIAIL collection showed the opposite skew in the new collection. These skews are within the expectations for random drift (Rockman and Kruglyak 2009).

### Implementation of high-throughput assays to measure fecundity and body size

As a scientific community, *C. elegans* researchers have few assays that can measure phenotypes for hundreds of independent strains quickly and reproducibly. Most phenotypic assays use a small number of strains in a large number of conditions because the majority of investigators only use the N2 genetic background. To understand the underlying allelic variants that contribute to complex traits, we require assays that can measure a large number of independent strains. Most chemical perturbations to an organism affect the number of offspring produced and the growth rate of animals. For these reasons, we created high-throughput assays to measure fecundity and body size for hundreds of independent strains quickly and accurately.

**Figure 1:**
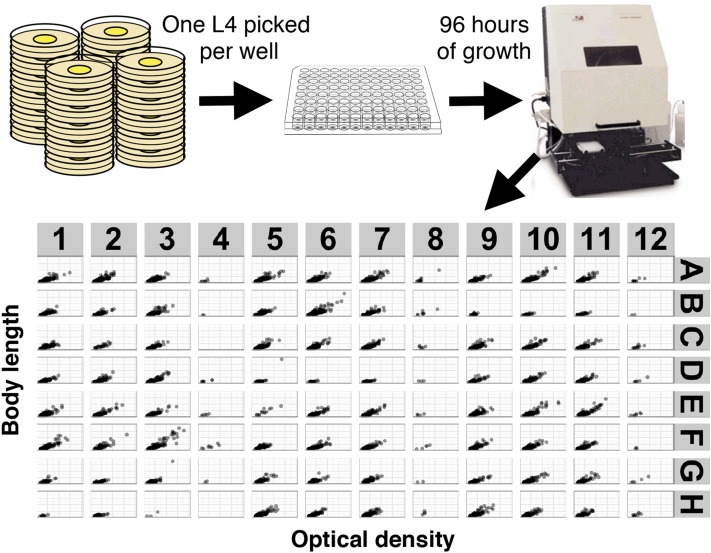
An overview of the high-throughput analysis pipeline is shown. Animals are grown on plates for five generations without starvation and then single hermaphrodites are picked to wells of a 96-well microtiter plate. The microtiter plate either has no compound (control) or paraquat added. Animals are grown for 96 hr at which point M9 solution with sodium azide is added to kill the nematodes and straighten them out. Each well is aspirated using the ReFLx module of the COPAS BIOSORT and animals are measured for length (time of flight), optical density (extinction), and three fluorescence measures. Raw data are read in, processed, and plotted using the COPASutils R package. Example length and optical density data are shown from one 96-wel plate with animals in every well except wells in columns 4, 8, and 12.

Previously, we found that measuring fecundity and body size by using standard laboratory agar plate-based methods would not work well for a large number of independent strains (Andersen *et al.* 2014). We aimed to develop a protocol with little human input for preparation of the assay and no human input in the measurement of the traits. For these reasons, we optimized a protocol in which researchers prepare the assay in microtiter plates and the COPAS BIOSORT platform (Union Biometrica) automatedly measures the traits of interest (Figure 1). In brief, hermaphrodites are singled into wells of a microtiter plate. Four days later, the clonal offspring of those single hermaphrodites are aspirated by the COPAS BIOSORT system outfitted with a ReFLx probe. The length (time of flight), optical density (extinction), and three fluorescence parameters of every nematode in every well are measured. From these measurements, the growth distribution among the offspring is determined. Additionally, the number of offspring per original hermaphrodite can be obtained by summing the total number of objects in each well of the microtiter plate. Raw data from the COPAS platform are read in, processed, and analyzed using a new R package called *COPASutils* (Shimko and Andersen 2014).

This liquid-handling platform worked well for our purposes, but parameters needed to be optimized to make it a useful quantitative assay. We prepared bacterial food in large batches and froze aliquots to make the food source reproducible across many months. Plates were housed in a humidity chamber with constant mixing and temperature. Our fecundities and growth rates are highly correlated with measurements from agar plates, indicating that *C. elegans* can thrive when grown in liquid conditions as long as bacterial food is distributed evenly. We found that intra-assay and inter-assay correlations for fecundity were substantially greater than fecundity assays using standard agar plate-based methods (Figure S4). These results indicate that these fecundity and body size assays are much more reproducible, more accurate, and higher throughput than standard *C. elegans* laboratory methods.

### High-throughput assays of fecundity and animal size generate rich multivariate traits with distinct underlying genetic causes

The raw data from the high-throughput assays described above provide a variety of measurements relating to fecundity and body size. We generated summary statistics from these measurements and treated the summary statistics as traits for quantitative trait mapping. For fecundity, we mapped the total number of animals in each well. For body size, the raw data provide five relevant parameters: time of flight, optical density, and red, yellow, and green fluorescence. Because fluorescence values were low, we did not analyze those data further. Time of flight is a direct measurement of body length, whereas the optical density parameter is influenced by body length, thickness, and composition. For each parameter, we calculated eight summary statistics: the average, variance, interquartile range, 10th, 25th, 50th (median), 75th, and 90th quantiles. Next, because body length is highly correlated with optical density (Figure S5), we also normalized the latter for body length and re-calculated the summary statistics. These normalized values better capture the contributions of body thickness and body composition to optical density. Additionally, the normalized optical density summary statistics are less correlated with length and optical density parameters. This data processing pipeline produces a single measurement of fecundity and 24 summary statistics related to body size (two raw parameters plus one normalized parameters times eight summary statistics). Finally, when chemical perturbations were measured along with paired controls (see next section), we performed an additional analysis where we used linear regression to reduce the contribution of the control trait to the perturbed trait.

**Figure 2:**
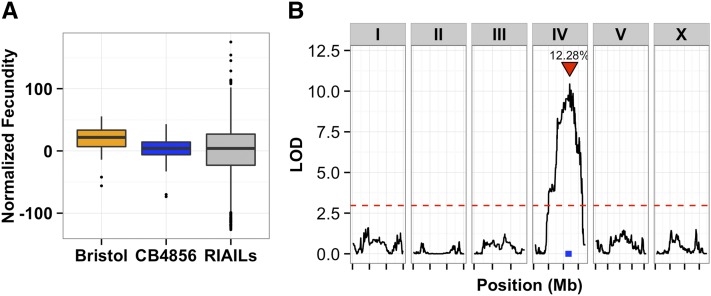
(A) Tukey box plots of N2 (orange), CB4856 (blue), and recombinant inbred advanced intercross lines (RIAILs; gray) normalized fecundity data. The total number of offspring from twenty replicates of each parent and 357 RIAILs were counted. The fecundities of the RIAILs show transgressive segregation. (B) Linkage mapping results of normalized fecundity in control conditions are shown with genomic position (Mb) on the x-axis and logarithm of odds (LOD) score on the y-axis. The tick marks on the x-axis denote every 5 Mb. Each chromosome is in its own box labeled on top. The dotted red line is the LOD threshold for 5% FDR obtained by permuting the phenotype data and mapping 1000 times. The red triangle denotes the peak QTL marker, and the blue bar shows the 95% QTL confidence interval. FDR, false discovery rate; QTL, quantitative trait loci.

We measured fecundity and all 24 body size traits for 357 of our N2xCB4856 RIAILs and a large number of QX1430 and CB4856 parent replicates. Many of the 24 body size traits were highly correlated with each other (Figure S5). For fecundity, 49.5% of trait variance in the population can be ascribed to genetic causes. Strains QX1430 and CB4856 differed in their fecundity, and the phenotypes of the RIAILs mostly ranged between the two parents (Figure 2A). This difference maps to a single significant QTL on chromosome IV, which explains 12% of the phenotypic variance (Figure 2B).

**Figure 3:**
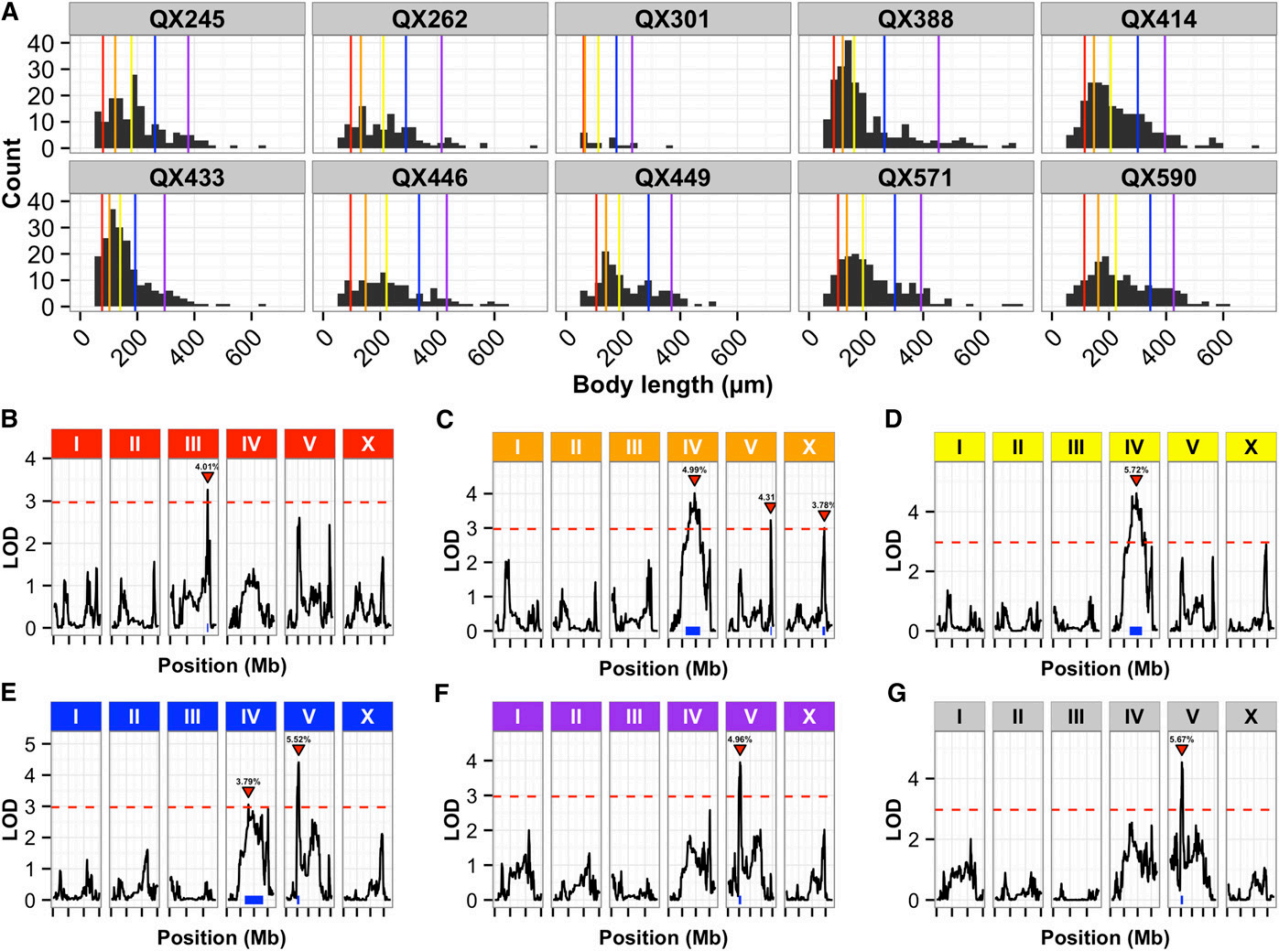
Summary statistics of body length map to different genomic regions implicating unique genetic variants. (A) Histograms of the length of every animal measured for 10 randomly chosen RIAILs are shown. The data for each RIAIL are in separate boxes with the strain name in bold above. The colored vertical lines denote population summary statistics, 10th quantile (red), 25th quantile (orange), median (yellow), 75th quantile (blue), 90th quantile (purple), and interquartile range (gray). The color of the vertical line matches the color of the chromosome label titles on top of the linkage mapping results for each trait. The genetic architectures differ when variation of size trait changes. B. Linkage mapping results of the 10th quantile of body length in control conditions are shown with genomic position (Mb) on the x-axis and logarithm of odds (LOD) score on the y-axis. The tick marks on the x-axis denote every 5 Mb. Each chromosome is in its own box labeled on top. The dotted red line is the LOD threshold for 5% FDR obtained by permuting the phenotype data and mapping 1000 times. The red triangle denotes the peak QTL marker, and the blue bar shows the 95% QTL confidence interval. (C) Linkage mapping results of the 25th quantile of body length in control conditions are shown with genomic position (Mb) on the x-axis and LOD score on the y-axis. The tick marks on the x-axis denote every 5 Mb. Each chromosome is in its own box labeled on top. The dotted red line is the LOD threshold for 5% FDR obtained by permuting the phenotype data and mapping 1000 times. The red triangle denotes the peak QTL marker, and the blue bar shows the 95% QTL confidence interval. (D) Linkage mapping results of the 50th quantile or median of body length in control conditions are shown with genomic position (Mb) on the x-axis and LOD score on the y-axis. The tick marks on the x-axis denote every 5 Mb. Each chromosome is in its own box labeled on top. The dotted red line is the LOD threshold for 5% FDR obtained by permuting the phenotype data and mapping 1000 times. The red triangle denotes the peak QTL marker, and the blue bar shows the 95% QTL confidence interval. E. Linkage mapping results of the 75th quantile of body length in control conditions are shown with genomic position (Mb) on the x-axis and LOD score on the y-axis. The tick marks on the x-axis denote every 5 Mb. Each chromosome is in its own box labeled on top. The dotted red line is the LOD threshold for 5% FDR obtained by permuting the phenotype data and mapping 1000 times. The red triangle denotes the peak QTL marker, and the blue bar shows the 95% QTL confidence interval. F. Linkage mapping results of the 90th quantile of body length in control conditions are shown with genomic position (Mb) on the x-axis and LOD score on the y-axis. The tick marks on the x-axis denote every 5 Mb. Each chromosome is in its own box labeled on top. The dotted red line is the LOD threshold for 5% FDR obtained by permuting the phenotype data and mapping 1000 times. The red triangle denotes the peak QTL marker, and the blue bar shows the 95% QTL confidence interval. G. Linkage mapping results of the variance of body length in control conditions are shown with genomic position (Mb) on the x-axis and LOD score on the y-axis. The tick marks on the x-axis denote every 5 Mb. Each chromosome is in its own box labeled on top. The dotted red line is the LOD threshold for 5% FDR obtained by permuting the phenotype data and mapping 1000 times. The red triangle denotes the peak QTL marker, and the blue bar shows the 95% QTL confidence interval. FDR, false discovery rate; QTL, quantitative trait loci; RIAIL, recombinant inbred advanced intercross lines.

**Figure 4:**
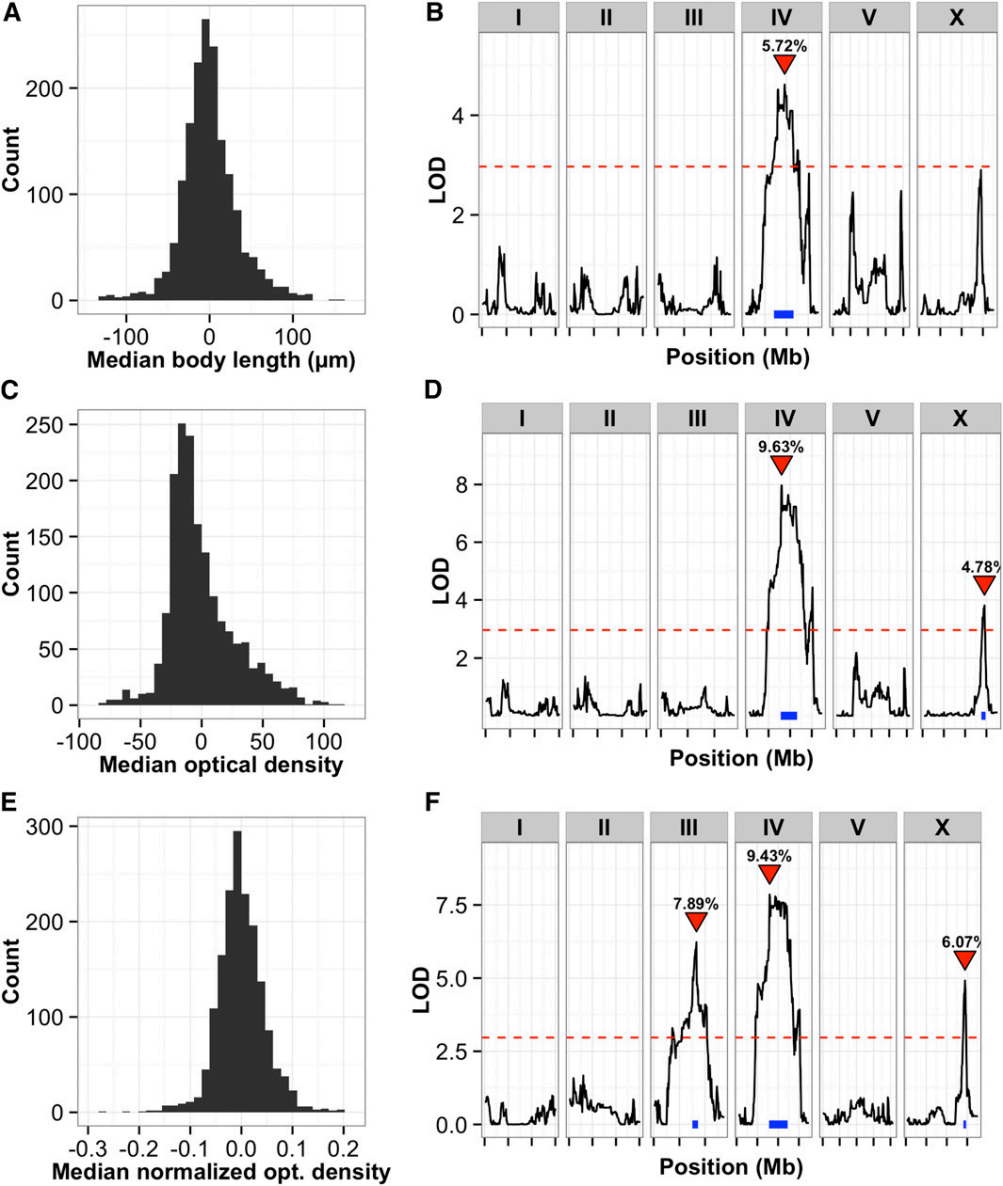
(A) Histogram of the normalized median body lengths of the RIAIL population in control conditions. Linear regression was used to reduce the effects of assay plate and plate position. (B) Linkage mapping results of median body length in control conditions are shown with genomic position (Mb) on the x-axis and logarithm of odds (LOD) score on the y-axis. The tick marks on the x-axis denote every 5 Mb. Each chromosome is in its own box labeled on top. The dotted red line is the LOD threshold for 5% FDR obtained by permuting the phenotype data and mapping 1000 times. The red triangle denotes the peak QTL marker, and the blue bar shows the 95% QTL confidence interval. (C) Histogram of the normalized median optical densities of the RIAIL population in control conditions. Linear regression was used to reduce the effects of assay plate and plate position. (D) Linkage mapping results of median optical density in control conditions are shown with genomic position (Mb) on the x-axis and LOD score on the y-axis. The tick marks on the x-axis denote every 5 Mb. Each chromosome is in its own box labeled on top. The dotted red line is the LOD threshold for 5% FDR obtained by permuting the phenotype data and mapping 1000 times. The red triangle denotes the peak QTL marker, and the blue bar shows the 95% QTL confidence interval. (E) Histogram of the median normalized optical densities (optical density of each animal divided by its length) of the RIAIL population in control conditions. Linear regression was used to reduce the effects of assay plate and plate position before normalizing for body length. (F) Linkage mapping results of median normalized optical density in control conditions are shown with genomic position (Mb) on the x-axis and LOD score on the y-axis. The tick marks on the x-axis denote every 5 Mb. Each chromosome is in its own box labeled on top. The dotted red line is the LOD threshold for 5% FDR obtained by permuting the phenotype data and mapping 1000 times. The red triangle denotes the peak QTL marker, and the blue bar shows the 95% QTL confidence interval. After normalizing for body length additional variation is revealed and maps to chromosomes III and X. FDR, false discovery rate; QTL, quantitative trait loci; RIAIL, recombinant inbred advanced intercross lines.

Next, we mapped each of the body size traits separately and obtained 47 significant QTL, which correspond to at least six distinct loci (Figure S6). Some loci were detected multiple times for sets of correlated traits. As an example of the type of data we observe, representative distributions of body lengths are shown (Figure 3A). Importantly, we find that the genetic architectures of traits change as different summary statistics for the same parameter are mapped. For example, as mappings of body length proceed from the 10th through the 90th quantiles, QTL appear and disappear on chromosomes III, IV, and V: the 10th quantile of body length mapped to a significant QTL on chromosome III (Figure 3B); in mappings of the 25th and 50th quantiles, a different QTL emerges (Figure 3, C and D); and in mappings of the 75th and 90th quantiles, yet a third QTL emerges (Figure 3, E and F). Thus, different QTL influence different subpopulations of the body length distribution. Because body length is a convenient proxy for developmental stage, these results likely indicate that distinct genetic loci influence different developmental stages of animals in a population. As another example of genetic architecture changing as different summary statistics are mapped, we find that QTL for optical density and body length largely overlap (as expected, given their tight correlation), but new QTL emerge when we map a normalized optical density trait (the optical density of each animal divided by its length, Figure 4). Taken together, these data show that using these high-throughput assays and our new RIAIL collection, we can rapidly and accurately map variants that control a variety of growth rate-related traits in *C. elegans*.

### *C. elegans* strains vary in their responses to the herbicide paraquat, and multiple QTL contribute to this difference

**Figure 5:**
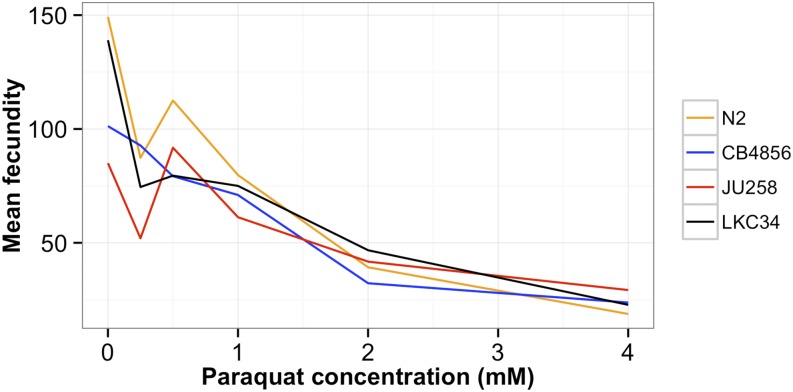
The normalized brood sizes of four strains in different concentrations of the herbicide paraquat are shown. N2 (orange), CB4856 (blue), JU258 (red), and LKC34 (black) have decreased growth rates and body sizes when exposed to increasing concentration of paraquat. The means of four replicates of each strain are shown.

We extended our high-throughput assays to map fecundity and body size traits in response to the chemical stressor paraquat. This widely used herbicide catalyzes the formation of reactive oxygen species and interferes with the electron transport chain in animal cells. Previous research in *C. elegans* indicated that the species is sensitive to paraquat (Ishii 2000) and that wild strains differ in responses to it (Vertino *et al.* 2011). To define an appropriate paraquat dose for our high-throughput assay, we measured fecundity and each of the body size traits for four divergent strains (Andersen *et al.* 2012) exposed to increasing concentrations of paraquat (Figure 5). The broad-sense heritability of fecundity and body size traits in paraquat conditions was 60% on average, indicating that the majority of phenotypic variance in the population can be attributed to genetic causes. Using these results, we chose 1.5 mM paraquat as an appropriate concentration for mapping paraquat responses in our RIAIL population because it had the largest difference between the parent strains along with high broad-sense heritability (0.56).

**Figure 6:**
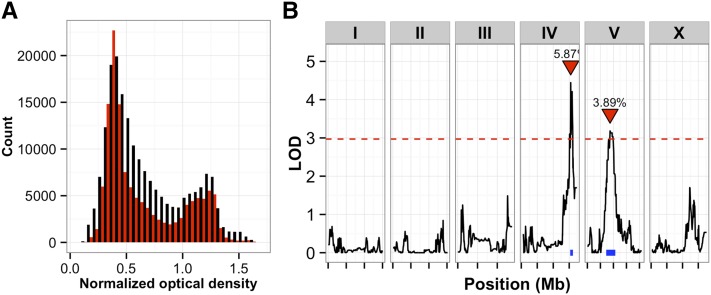
(A) Histograms of mean normalized optical density in control (black) and in paraquat (red) conditions for the 357 RIAILs are shown. (B) Linkage mapping results of the residual normalized mean optical density in paraquat conditions are shown with genomic position (Mb) on the x-axis and logarithm of odds (LOD) score on the y-axis. The tick marks on the x-axis denote every 5 Mb. Each chromosome is in its own box labeled on top. The dotted red line is the LOD threshold for 5% FDR obtained by permuting the phenotype data and mapping 1000 times. The red triangle denotes the peak QTL marker, and the blue bar shows the 95% QTL confidence interval. FDR, false discovery rate; QTL, quantitative trait loci; RIAIL, recombinant inbred advanced intercross lines.

Next, using our high-throughput assays, we exposed 357 of the 359 N2xCB4856 RIAILs and replicates of the parent strains to 1.5 mM paraquat and measured fecundity and each of the body size traits. As expected, fecundity was reduced after paraquat exposure, as compared to control conditions (data not shown). For QTL mapping of paraquat-specific responses, we used linear regression to reduce the contributions of control conditions to the paraquat phenotype data and then mapped the residual traits in the same way as the control traits. Nine additional QTL were detected that represent two distinct paraquat-specific genomic regions on chromosomes IV and V (Figure 6). These two loci each control approximately 5% of the phenotypic variance for traits related to optical density (Figure 6B). Moreover, these loci do not overlap QTL detected in control conditions (Figure S7), indicating that separate loci control growth in paraquat beyond any differences observed in control growth conditions. Finally, when considering QTL detected in any condition, we found that the proportion of phenotypic variance explained by each QTL was on average 5% (Figure S8). When considered additively for any particular trait, the detected QTL explained 10% of the phenotypic variance on average. Thus, our high-throughput assays and this panel of new strains enabled the detection of QTL with small phenotypic effects affecting a variety of traits.

## Discussion

To supplement an existing collection of recombinant inbred lines between the laboratory strain (N2) and a wild strain from Hawaii (CB4856), we created an additional 359 RIAILs, more than doubling the total size of this resource. We also optimized high-throughput assays to measure fecundity and size parameters for populations of nematodes. These assays enable studies of quantitative trait variation for a large number of fitness-related traits. We mapped these traits in normal growth conditions and also after exposure to the herbicide paraquat. These mappings identified 56 QTL, representing eight distinct loci for sets of correlated traits. Together, these resources constitute a powerful new experimental platform to study phenotypic effects of diverse treatments in a large number of independent strains in a reproducible and scalable manner.

### Persistence of allele frequency skews in the new RIAIL panel

This new collection of RIAILs was constructed using the same cross design as the previous collection (Rockman and Kruglyak 2009). However, we used strain QX1430 instead of N2 to eliminate the *peel-1zeel-1* incompatibility (Seidel *et al.* 2011, 2008) and the large phenotypic effects of *npr-1* (Andersen *et al.* 2014). Surprisingly, we still see allele frequency skews on chromosome I and other chromosomes, suggesting that subtle incompatibilities still exist. The remaining skew on chromosome I may have been caused by genes other than the *peel-1*; alternatively, the transposon insertion into *peel-1* might not cause complete loss of gene function. We carried out our crosses at 20°, rather than 25°, which could have allowed different alleles to have been selected during the RIAIL panel construction. Larger RIAIL or NIL panels might allow us to map these subtle incompatibilities further.

### Multivariate data uncover multiple contributors to growth rate

The high-throughput phenotyping platform generates a large amount of data from populations of independent strains. Along with counting each animal in every well of a microtiter plate, the length, optical density, and three fluorescent parameters of each individual are recorded. Summary statistics from distributions of these parameters, like quantiles, means, interquartile ranges, and variances, are calculated. Many of these traits are correlated with each other. For example, small body size as indicated by the 10th quantile of length is correlated with the 10^th^ quantile of optical density. However, we found that different summary statistics recorded for the RIAILs sometimes map to different regions of the genome. The population of animals derived from the hermaphrodite mother varies in life stage from early larval animals to young adults. In control conditions without any paraquat applied, we find that the summary statistics that describe distributions from the RIAILs are different, indicating that growth rates (as measured by the length of offspring or optical density) vary in our strain population. Other researchers have used these size parameters to fit models of growth (Boyd *et al.* 2009; Smith *et al.* 2009). Interestingly, our data suggest that life-stage progression is controlled by different genetic mechanisms because we often identify distinct QTL for small size *vs.* large size. Our results indicate that the COPAS BIOSORT platform is a powerful tool to obtain multivariate data from large numbers of independent strains and observe relatively small phenotypic effects.

### Comparison with previous QTL studies in *C. elegans*

Our mapping results recapitulate and expand upon previous QTL studies in *C. elegans*. Previously, we mapped adult body size to two QTL on chromosomes V and X (Andersen *et al.* 2014). The QTL on chromosome V also was detected in this study as a regulator of body length but only when looking at larger animals in the distribution (75th and 90th quantiles), suggesting that this genetic locus may influence body length in later developmental stages. As expected, we did not identify the QTL on chromosome X because the new mapping population no longer varies for the *npr-1* gene. We also mapped fecundity to two QTL on chromosomes II and X (Andersen *et al.* 2014). Like the previous mapping result, the lack of variation at *npr-1* likely eliminates the QTL on chromosome X. The other QTL on chromosome II was at the threshold of detection and could have been falsely discovered in our previous analysis. In this study, we identified a QTL on chromosome IV for fecundity that was not identified in the earlier analysis. Because a QTL with this effect size is near the level of detection in the previous study, we believe that it was possibly a false negative. In two independent crosses—BergeracxRC301 and CB4857xRC301—a QTL was detected just right of center on chromosome IV that contributed to variation in longevity (Ayyadevara *et al.* 2003, 2001). This QTL also contributed to variation in responses to paraquat (Vertino *et al.* 2011). An overlapping genomic region was detected in our current study as a regulator of optical density in paraquat conditions. Further investigation will determine whether these QTL are separate loci and whether the genomic region we identified also affects variation in longevity. Using imaging, investigators scored another collection of N2xCB4856 recombinant strains for variation in body size and found a QTL on chromosome IV but did not detect QTL overlapping with our results (Kammenga *et al.* 2007). This discrepancy is likely caused by differences in the body size assay. Notably, recombinant inbred line panels constructed using the N2 strain or derivatives likely harbor numerous laboratory-derived alleles in addition to *npr-1* and care should be taken while interpreting evolutionary conclusions.

This new collection of RIAILs will drastically increase statistical power beyond the current set of N2xCB4856 RIAILs. When phenotyped as a complete set, the combined panel should allow researchers to detect QTL that individually explain as little as 2% of the phenotypic variance for quantitative traits. When combined with our new high-throughput phenotyping assays, the panel will enable mapping of multivariate phenotype measurements for responses to any aqueous compound, altered bacterial food sources, or biotic stresses. We believe that these developments increase the power of *C. elegans* quantitative genetics in substantial ways.
